# Association between the social exposome and vascular brain injury in brain bank donors

**DOI:** 10.1002/alz.090924

**Published:** 2025-01-09

**Authors:** Sarah A. Keller, W. Ryan Powell, William R. Buckingham, Amanda DeWitt, Barbara B. Bendlin, Amy J.H. Kind

**Affiliations:** ^1^ Center for Health Disparities Research, University of Wisconsin School of Medicine and Public Health, Madison, WI USA; ^2^ Department of Medicine, Geriatrics Division, University of Wisconsin School of Medicine and Public Health, Madison, WI USA; ^3^ Wisconsin Alzheimer’s Disease Research Center, University of Wisconsin School of Medicine and Public Health, Madison, WI USA

## Abstract

**Background:**

The social exposome, all social‐environmental exposures accumulated over the life course, could partially explain Alzheimer’s disease and other dementias (ADRD) disparities. Measurement and linkage of life course social exposome metrics to biological samples may inform opportunities for intervention. While there is increasing emphasis on the life course social exposome in ADRD research, there is little consensus on its measurement. Vascular brain injury (VBI) is a measure of changes in the cerebral blood vessels associated with increased risk of ADRD, but the extent to which social exposures are associated with VBI is unknown.

**Method:**

We evaluated the association between life course social exposome measures and the VBI burden acrosstwo Alzheimer’s Disease Research Center (ADRC) brain banks. Geocoded life course donor addresses were linked to time‐concordant national Area Deprivation Index (ADI) rankings, with greater ADI denoting greater county‐level disadvantage. Life course social exposome was examined separately as 1, a cumulative measure of the number of years spent above the population median life course ADI, and 2, the change in ADI from youth to adulthood. The outcome was the presence of any of four VBI indicators: infarcts, microinfarcts, hemorrhages, and white matter rarefaction. The association of life course social exposome to VBI burden was evaluated via logistic regression adjusting for sex and age at death.

**Result:**

This sample contained 740 donors from two brain banks. The population median ADI was 7.08 (IQR, 5.5) and the mean number of years spent above this threshold was 19.95 (SD, 20.89). More years living in a county above the population median was associated with increased VBI burden (OR, 1.04, 95% CI, 1.03‐1.05). A median ADI which increased (OR, 3.67, 95% CI, 3.65‐3.70) or remained stably high between youth and adulthood (OR, 3.08, 95% CI, 3.01‐3.14) resulted in increased odds of VBI burden.

**Conclusion:**

This study shows promise for clarifying the relationship between the social exposome and ADRD brain pathology post‐mortem. We found associations between higher life course ADI (greater disadvantage) and VBI burden. Identifying dosages and timing of life course exposures critical for development of VBI allows for future intervention to reduce disparities in ADRD.

